# Ectopically Expressed Variant Form of Sperm Mitochondria-Associated Cysteine-Rich Protein Augments Tumorigenicity of the Stem Cell Population of Lung Adenocarcinoma Cells

**DOI:** 10.1371/journal.pone.0069095

**Published:** 2013-11-11

**Authors:** Akari Takahashi, Yoshihiko Hirohashi, Toshihiko Torigoe, Yasuaki Tamura, Tomohide Tsukahara, Takayuki Kanaseki, Vitaly Kochin, Hiroshi Saijo, Terufumi Kubo, Munehide Nakatsugawa, Hiroko Asanuma, Tadashi Hasegawa, Toru Kondo, Noriyuki Sato

**Affiliations:** 1 Department of Pathology, Sapporo Medical University School of Medicine, Chuo-ku, Sapporo, Japan; 2 Department of Surgical Pathology, Sapporo Medical University School of Medicine, Chuo-ku, Sapporo, Japan; 3 Department of Stem Cell Biology, Hokkaido University Graduate School of Medicine, Kita-Ku, Sapporo, Japan; 4 Cancer Diagnosis Laboratory, Japan Science and Technology Agency Innovation Plaza Hokkaido, Japan Science and Technology Agency, Kita-Ku, Sapporo, Japan; Deutsches Krebsforschungszentrum, Germany

## Abstract

Cancer stem-like cells (CSCs)/cancer-initiating cells (CICs) are defined as a small population of cancer cells that have self-renewal ability, differentiation ability and high tumor-initiating ability. CSCs/CICs are resistant to cancer therapies including chemotherapy and radiotherapy. Therefore, CSCs/CICs are thought to be responsible for cancer recurrence and distant metastasis after treatment. However, the molecular mechanisms of CSCs/CICs are still elusive. In this study, we isolated CSCs/CICs as side population (SP) cells from lung carcinoma, colon carcinoma and breast carcinoma cells and analyzed the molecular mechanisms of CSCs/CICs. cDNA micro-array screening and RT-PCR analysis revealed that sperm mitochondria-associated cysteine-rich protein (SMCP) is ectopically expressed in SP cells. 5′-Rapid amplification of cDNA end (RACE) analysis revealed that the SMCP transcript in SP cells was a variant form (termed vt2) which is composed from only one exon. SMCP vt2 was detected in only cancer cells, whereas the wild-type (vt1) form of SMCP was expressed in the testis. SMCP was shown to have a role in tumor initiation by SMCP overexpression and SMCP knockdown using siRNAs in lung cancer cells. Taken together, the initiation results indicate that an ectopically expressed variant form of SMCP has a role in tumor initiation of CSCs/CICs and that the variant form of SMCP might be a novel CSC/CIC marker and a potential and promising target of CSC/CIC-targeting therapy.

## Introduction

Recent progress in cancer research has revealed that cancers are composed of morphologically and phenotypically heterogenous malignant transformed cells, and only a small population of cancer cells have tumor-initiating ability when transplanted into immune-deficient mice (cancer stem cell hypothesis) [1], [2]. These cells with high tumorigenicity are called cancer stem-like cells (CSCs)/cancer-initiating cells (CICs). In subsequent works, CSCs/CICs were shown to be resistant to chemotherapies and radiotherapies [3], [4]. Therefore, CSCs/CICs are thought to be responsible for disease recurrence after treatments and for distant metastasis, which make the prognosis poor. Thus, eradication of CSCs/CICs is essential for curing cancer.

CSCs/CICs have been isolated by several methods [5], including (1) use of cell surface markers such as CD44^+^CD24^−^ [6], CD133^+^ [7], [8] and CD166^+^ [9], (2) side population (SP) analysis [10], (3) ALDEFLUOR assay [11], and (4) spheroid culture [12]. Using these methods CSCs/CICs have been successfully isolated from acute myeloid leukemia [13]–[15] breast cancer [6], lung cancer [16], colon cancer [7], [8], [17], brain tumors [10], [18]–[21], prostate cancer [22]–[24], pancreas cancer [25], [26], liver cancer [27] and melanoma [28]. However, these markers are merely surrogate markers, and sometimes the expression of CSC/CIC markers did not indicate highly tumorigenic CSCs/CICs [29], [30]. Therefore, further investigations of CSC/CIC markers and molecular mechanisms of CSC/CIC are needed for the establishment of CSC/CIC targeting therapy.

In this study, we investigated molecular aspects of highly tumorigenic SP cells derived from lung, colon and breast cancer cells and found that a splicing variant form of sperm mitochondria-associated cysteine-rich protein (SMCP), which is expressed in normal testis tissues, is ectopically expressed in SP cells and that SMCP has a role in the tumor-initiating ability of SP cells. These findings indicate that SMCP might be a novel CSC/CIC marker and a potential target for CSC/CIC targeting therapy.

## Materials and Methods

### Ethics statement

Mice were maintained and experimented on in accordance with the guidelines of and after approval by the Committee of Sapporo Medical University School of Medicine, Animal Experimentation Center under permit number 10-032. Any animal found unhealthy or sick was promptly euthanized. All studies were approved by Institutional Review Boards (IRB) of Kushiro City General Hospital. We obtained written informed consent from all patients according to the guidelines of the Declaration of Helsinki.

### Tissue samples

All studies were approved by Institutional Review Boards (IRB) of Kushiro City General Hospital. Three pairs of lung cancers and adjacent non-neoplastic lung tissues were obtained from surgically resected tissues removed at Kushiro City General Hospital. The histological types of the three cancer tissues were: case #1 and 2, adenocarcinoma; case #3 and 4, squamous cell carcinoma; and case #5, large cell carcinoma.

### Cell culture

Lung adenocarcinoma cell line LHK2 was established in our laboratory [31]. Lung small cell carcinoma cell line Lc817 was purchased from the Japanese Cancer Research Resources Bank (Osaka, Japan). Human breast adenocarcinoma cell line MCF7, human lung adenocarcinoma cell line A549 and human embryonal kidney cell line HEK293T were purchased from ATCC (Manassas, VA, USA). Colon adenocarcinoma cell line SW480 [32] was a kind gift from Dr. K. Imai (Sapporo, Japan). These cell lines were cultured in Dulbecco's Modified Eagle's Medium (DMEM) (SIGMA-ALDRICH, St. Louis, MO, USA) with 10% heat-inactivated fetal bovine serum (FBS; MP Biomedicals, Irvine, CA, USA) at 37°C in a humidified atmosphere containing 5% CO_2_. The retrovirus packaging cell line PLAT-A was kindly provided by Dr. T. Kitamura (Tokyo, Japan) [33]. PLAT-A was maintained in DMEM containing 10% FBS, 1 µg/mL puromycin (SIGMA-ALDRICH), and 10 µg/mL blasticidin (SIGMA-ALDRICH).

### Side population (SP) assay

Side population (SP) analysis was performed as described previously with some modifications [34], [35]. The cells were stained with Hoechst33342 dye (Lonza, Walkersville, MD, USA) at final concentrations of 5 µg/ML for LHK2 and SW480 cells and 2 µg/ml for MCF7 cells with or without Verapamil (SIGMA-ALDRICH), an inhibitor of ABC transporters, at the concentration of 75 µM. The cells were incubated at 37°C for 90 min with continuous shaking. Stained cells were analyzed by a FACS Aria II (BD Biosciences, San Jose, CA, USA). The Hoechst 33342 dye was excited at 357 nm and its fluorescence was analyzed using dual wavelengths (blue, 402–446 nm; red, 650–670 nm).

### RNA extraction and reverse transcription-PCR analysis

Isolation of RNA and RT-PCR analysis were performed as described previously [36]. Human Multiple Tissue cDNA Panels I and II (TAKARA BIO INC., Otsu, JAPAN) and the Human Fetal Multiple Tissue cDNA Panel (TAKARA BIO INC.) were used as templates of normal adult and fetal tissue cDNA. PCR amplification was performed in 20 µl of PCR mixture containing 1 µl of cDNA mixture, 0.5 µl of Taq DNA polymerase (QIAGEN) and 4 pmol of primers. The PCR mixture was initially incubated at 94°C for 2 min, followed by 35 cycles of denaturation at 94°C for 15 sec, annealing at 58°C for 30 sec, and extension at 72°C for 30 sec. Primer pairs used for RT-PCR analysis were 5′-TGGAGAAGGAGAAGCTGGAGCAAAA-3′ and 5′-GGCAGATGGTCGTTTGGCTGAATA-3′ for *POU5F1* with an expected PCR product size of 163 bps, 5′- GCTGAGATGCCTCACACGGAG-3′ and 5′- TCTGTTTCTTGACCGGGACCTTGTC-3′ for *NANOG* with an expected PCR product size of 161 bps, 5′- TGTGTGACCAGACAAAACACAG -3′ and 5′- GTTGGGCTCAGACTCCATGT -3′ for *SMCP* with an expected PCR product size of 249 bps, 5′- TCACTAGGCTGCTGAGGAAGA -3′ and 5′- CTGGGCAGCATTTACTGTGT -3′ for *SMCP-variant1* with an expected PCR product size of 152 bps, 5′- TTCAGGaAGCGTGTGACAGT -3′ and 5′- CTGGGCAGCATTTACTGTGT -3′ for *SMCP-variant2* with an expected PCR product size of 624 bps, and 5′-ACCACAGTCCATGCCATCAC-3′ and 5′-TCCACCACCCTGTTGCTGTA-3′ for *glyceraldehyde-3-phosphate dehydrogenase (GAPDH)* with an expected product size of 452 bps. *GAPDH* was used as an internal control. PCR amplification for *SOX2* was performed with PrimeSTAR HS DNA polymerase (TAKARA BIO INC.). The PCR mixture was initially incubated at 98°C for 2 minutes, followed by 35 cycles of denaturation at 98°C for 15 seconds, and annealing and extension at 68°C for 30 seconds. *SOX2* expression was detected using sense primer 5′-CATGATGGAGACGGAGCTGA-3′ and antisense primer 5′-ACCCCGCTCGCCATGCTATT-3′ with an expected PCR product size of 420 bp.

### Quantitative real-time PCR analysis

Quantitative real-time PCR was performed using the ABI PRISM 7000 Sequence Detection System (Applied Biosystems, Foster City, CA) according to the manufacturer's protocol. SOX2 primer and probe was designed by the manufacturer (TaqMan Gene expression assays; Applied Biosystems). Thermal cycling was performed using 40 cycles of 95°C for 15 seconds followed by 60°C for 1 min. Each experiment was done in triplicate, and normalized to the *GAPDH* gene as an internal control.

### Microarray analyses

We used the commercially available amino-allyl RNA amplification Kit ver,2 (High Yield Type) (SIGMA-ALDRICH). Purified total RNA (3 µg) was reverse-transcribed to generate double-stranded cDNA using an oligo dT T7 promoter primer and reverse transcriptase. Next, cRNA was synthesized using T7 RNA polymerase, which simultaneously incorporated Cy3- or Cy5-labeled cytidine triphosphate. During this process, the samples of SP cells were labeled with Cy5, whereas the non-SP cells were labeled with Cy3 as control cells. Quality of the cRNA was again checked using the Nano Drop. Cy3-labeled cRNA and Cy5-labeled cRNA were combined and then fragmented in a hybridization cocktail (SIGMA-ALDRICH). Then the labeled cRNAs were hybridized to a 60-mer probe oligonucleotide microarray (Panorama Human Micro Array, SIGMA-ALDRICH) and incubated for 20 h ours at 50°C. The fluorescent intensities were determined by a Genepix 4000B Microarray Scanner (Axon, US). We performed the samples of SP cells were labeled with Cy3 whereas the non-SP cells were labeled with Cy5. Microarray raw data and processed data have been deposited in a ArrayExpress database (http://www.ebi.ac.uk/arrayexpress/, Accession Number: E-MEXP-3913).

### 5′-Rapid amplification of cDNA ends (5′-RACE) and 3′-RACE

5′ Rapid amplification of cDNA ends (5′-RACE) and 3′-RACE were carried out using total RNA isolated from Lc817 cells to identify the transcriptional start site of the SMCP gene in the cancer cell lines, following the protocol of manufacturer (TAKARA BIO INC.). Briefly, 1 µg of total RNA was utilized to generate either 5′ or 3′ RACE Ready cDNA products with specific primers and reagents provided by the kit in the presence of Moloney Murine Leukemia Virus (MMLV) reverse transcriptase. Generation of the 5′-RACE fragment was performed as described in the protocol. In brief, 50 µl PCR reaction mixture consisting of 2.5 µl of 5′-RACE-Ready cDNA, 5 µl of 10×Universal Primer A Mix (UPM), 1 µl of 10 µM Gene Specific Primer 1 (GSP1) and 41.5 µl of Master Mix (TAKARA BIO INC.) was prepared. Positive and negative controls were prepared according to the protocol of the manufacturer. GSP1 primer, 5′- ATGGCCCCAGGGACTTCTTCTTTGT-3′, was designed on the basis of nucleotide sequences of SMCP CDS. Nested PCR reaction was done by using diluted 1^st^ PCR production, 1 µl nested universal primer A (NUP) and 1 µl nested GSP1 primer, located upstream of the GSP1 primer, 5′-TGGCCTGGACTCATTTTGTGGGCTA-3′, instead of the UPM primer and GSP1 primer. In order to generate the 3′ region fragment, 3′-RACE was performed by using a 50 µl PCR reaction mixture cosisting of 2.5 µl of 5′-RACE-Ready cDNA, 5 µl of 10×UPM, 1 µl of 10 uM GSP2, 5′-CTGTGGTTTGGAGACCAAGCCTGAA-3′, and 41.5 µl of master mix. In nested PCR, 1 µl nested GSP2, 5′- ATGGAGTCTGAGCCCAACTCACCGCAAA-3′, was used. All PCR products were purified and sequences were confirmed by DNA sequencing.

### Plasmid construct

The cDNA of human SMCP CDS was isolated from a human testis cDNA library (TAKARA BIO INC.) by PCR, meanwhile splicing variant2 was isolated from lung small cell carcinoma cell line Lc817 by PCR. The SMCP CDS and splicing variant2 was constructed into a pcDNA3.1(+) vector (Life Technologies, Carlsbad, CA, USA), respectively.

### Western blotting

Western blotting was performed as described previously [37]. Briefly, HEK293T cells were transfected with SMCP CDS plasmid and splicing variant2 plasmid and then lysed in 1 mL of SDS sample buffer. Anti-SMCP rabbit polyclonal antibody (custum ordered, Life Technologies) was used at 200-times dilution. Anti-β-Actin mouse monoclonal antibody (Sigma, St. Louis, MO, USA) was used at 2000-times dilution.

### Mice and xenograft transplantation

Non-obese diabetic-severe combined immunodeficient (NOD/SCID) mice were purchased from Sankyo Laboratory Co. Ltd. (Tsukuba, Japan). Studies were performed with approval of the Animal Experiment Ethics Committee of Sapporo Medical University (Sapporo, Japan).

Various numbers of SP and non-SP cells or LHK2-SMCP and LHK2-Mock cells were suspended in a PBS and Matrigel (BD Biosciences) mixture and transplanted to NOD/SCID mice (female, 4–6 wk of age) under anesthesia. SP and non-SP cells or LHK2-SMCP and LHK2-Mock cells were implanted into the s.c. space on the left and right sides of the back of recipient mice, respectively. Tumor formation was observed weekly for 10–13 weeks. The transplantation assays were performed in accordance with institutional guidelines for the use of laboratory animals.

### Generation of GFP-SMCP cell line

The cDNA of human SMCP was isolated from a human testis cDNA library (TAKARA BIO INC.) by PCR. The full-length SMCP was constructed into a pcDNA3.1 (+) vector (Life Technologies, Grand Island, NY, USA) fused with green fluorescent protein (GFP) gene. LHK2 cells were transfected with SMCP-GFP fusion expression plasmid by Lipofectamine 2000 (Life Technologies) and cultured in DMEM supplemented with 10% of FBS and 800 µg/mL Geneticin (Life Technologies).

### Generation of stable cell line overexpressing SMCP

Introduction of DNA into LHK2 cells was performed by a retrovirus-mediated method [33]. Briefly, the culture supernatant of PLAT-A cells transduced with the pMXs-based retrovirus vectors containing FLAG-epitope-tagged SMCP, was added onto LHK2 cells in the presence of 8 µg/mL of polybrene (SIGMA-ALDRICH) for 48 hrs. Two days later, the medium was replaced with a fresh medium containing 1 µg/ml puromycin for LHK2 cells to select the infected cells. The mRNA expression of SMCP in the puromycin-resistant sub-line was confirmed by RT-PCR.

### Small interfering RNA transfection

SMCP small interfering RNA (siRNA) duplexes were designed and synthesized using the BLOCK-it RNAi designer system (Life Technologies). The oligonucleotide encoding SMCP siRNA1 was 5′- CCCTTAACATGGAGTCTGAGCCCAA -3′, and that encoding siRNA2 was 5′- GCAATCAATGCTGCCCACCACAGCA -3′. Cells were seeded at 50% confluence, and transfections were carried out using Lipofectamine RNAiMAX (Life Technologies) in Opti-MEM according to the manufacturer's instructions.

### Confocal imaging

LHK2-GFP-SMCP cells were seeded on glass coverslips for 24 hr. The cells were then fixed with 4% paraformaldehyde, permeabilized with 0.1% Triton X-100, and then blocked with 10% goat serum for 40 min. The cells were first stained with MitoTracker Red 580 Dye (Life Technologies) for detecting mitochondria at RT for 20 min and washed. Following this, they were stained with DAPI (Life Technologies) at RT for 5 min and mounted. Each sample was visualized using an LSM510 META ConfoCor3 (Zeiss, Oberkochen, Germany), and images were captured and analyzed using the Zeiss LSM Image Browser (Zeiss).

### Statistical analysis

Data are presented as means ± SD. Differences in variables were assessed using Student's t-test. *P*<0.05 was considered significant.

## Results

### Isolation of CSCs/CICs as SP cells in human lung, breast and colon carcinoma cell lines

**Figure 1:**
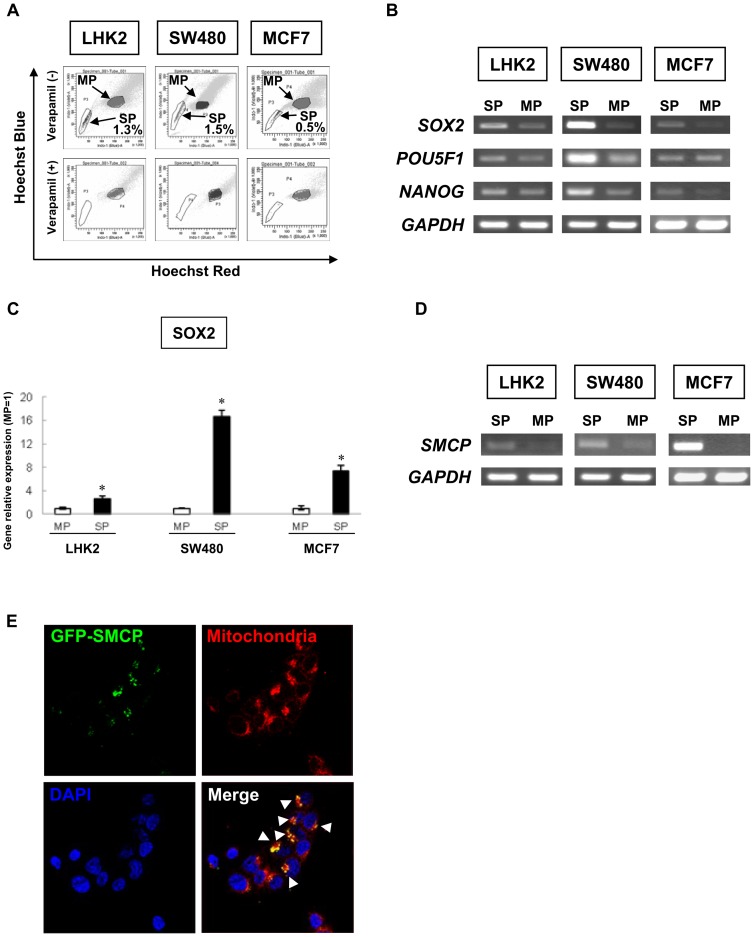
Isolation of a novel CSC/CIC molecule, SMCP. **A. Isolation of SP cells from LHK2, MCF7 and SW480 cells.** Lung (LHK2), colon (SW480) and breast (MCF7) cancer cells were stained with Hoechst_33342_ dye in the absence (upper panel) or presence (lower panel) of verapamil. All cell lines were analyzed by a cell sorter. **B. Expression profiles of stem cell markers in SP cells and MP cells.** Expression of stem cell markers (*SOX2*, *POU5F1* and *NANOG*) in SP cells and MP cells derived from LHK2, SW480 and MCF7 cells was determined. *GAPDH* was used as an internal positive control. **C. Quantitative real-time PCR analysis of SOX2 mRNA expression in SP and MP cells.** The MP cells were used for the control, which was set as 1.0. Data are expressed as mean + SD of relative values compared to MP cells. **D. Expression of** ***SMCP*** **in SP and MP cells.** Expression of *SMCP* in SP cells and MP cells derived from LHK2, SW480 and MCF7 cells was determined. **E. SMCP is localized in mitochondria.** LHK2 cells transfered with GFP-fused SMCP gene were fixed and stained with Mitotracker Red followed by DAPI and then visualized by laser confocal microscopy (Magnification, ×200). Green indicates GFP-fused SMCP. Red indicates mitochondria. Blue indicates nucleus.

SP cells derived from LHK2, SW480 and MCF7 cells are enriched with CSCs/CICs [35], [38], [39]. We therefore isolated SP cells from LHK2, SW480 and MCF7 cells as CSC/CIC sources. The cells were stained with Hoechst 33342 dye and analyzed with a FACS Aria II flow cytomerter. The rations in LHK2, SW480 and MCF7 cells of SP cells were 1.3%, 1.5% and 0.5%, respectively (Figure 1A). Greater tumor-initiating ability of SP cells was confirmed by injecting NOD/SCID mice with the cells (Table 1) [38]. To address the expression levels of stem cell markers in SP cells, we performed RT-PCR analysis. *SOX2*, *POU5F1* and *NANOG* were expressed in SP cells derived from LHK2, SW480 and MCF7 cells at higher levels than those in MP cells (Figure 1B). Moreover, we evaluated three mRNA transcripts with quantitative real-time PCR. As shown in figure 1C, *SOX2* mRNA was significantly overexpressed in SP cells compared with MP cells. The results indicated that these SP cells had molecular properties similar to those of iPS cells [40].

**Table 1:** Tumor-initiating ability of SP and MP cells.

MCF7		Cell numbers of injection into NOD/SCID mice			
Day 91 post injection		1.5×10^1^	1.5×10^2^	1.5×10^3^	1.5×10^4^
SP cells	Incidence	1/1	1/1	1/1	n.d.
	Volumes (mm^3^)	0.5	683.6	288.8	-
MP cells	Incidence	0/1	0/1	1/1	1/1
	Volumes (mm^3^)	-	-	165.5	691.9

SW480		Cell numbers of injection into NOD/SCID mice			
Day 42 post injection		1.0×10^1^	1.0×10^2^	1.0×10^3^	1.0×10^4^
SP cells	Incidence	1/3	1/3	3/3	3/3
	Volumes (mm^3^)	40	720	1225±571.7	7167±2020
MP cells	Incidence	0/3	1/3	3/3	3/3
	Volumes (mm^3^)	-	50	281±129	2789±1910

Incidence indicates the number of tumor formation/number of injections.

Tumor volumes are mean±S.D.

### Sperm mitochondria-associated cysteine-rich protein (SMCP) is preferentially expressed in SP cells

To address the molecular mechanism of SP cells, we performed gene chip microarray screening using total RNA from LHK2 SP cells and LHK2 MP cells (Table S1). We found that *SMCP* gene oligo reactive mRNA was overexpressed in LHK2 SP cells compared with the expression level in LHK2 MP cells. RT-PCR analysis revealed that *SMCP* mRNA was overexpressed in SP cells derived from LHK2, SW480 and MCF7 cells compared to the expression levels in MP cells (Figure 1D). Since the SMCP protein was reported to be expressed in sperm mitochondria [41], the cellular localization of SMCP in cancer cells was investigated using a GFP-fused SMCP gene. Co-localization of GFP-fused SMCP and a mitochondria marker (MitoTracker) was observed in LHK2 cells with overexpressed GFP-SMCP gene, indicating that ectopically expressed SMCP protein is also localized in mitochondria (Figure 1E).

### Expression profiles of SMCP in normal tissues and cancer cells

**Figure 2:**
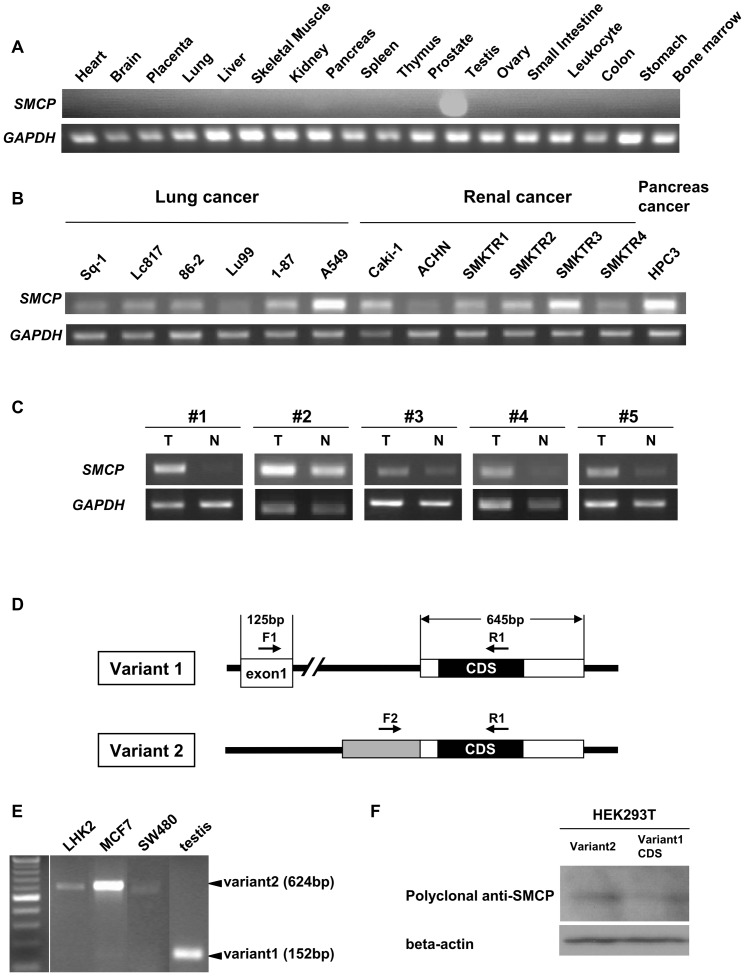
Expression profiles of SMCP and identification of a novel variant form of SMCP. **A. SMCP expression profiles in normal human organs.** SMCP mRNA expression in normal tissues (heart, brain, placenta, lung, liver, skeletal muscle, kidney, pancreas, spleen, thymus, prostate, testis, ovary, small intestine, leukocyte, colon, stomach and bone marrow) was investigated by RT-PCR. cDNAs were obtained from TAKARA BIO INC. (Human Multiple Tissue cDNA Panels I and II). *GAPDH* was used as an internal positive control. **B. SMCP expression profiles in human cancer cells.** SMCP mRNA expression in lung cancer line cells (Sq-1, Lc817, 86-2, Lu99, 1-87, A549), renal cancer line cells (Caki-1, ACHN, SMKTR1, SMKTR2, SMKTR3, SMKTR4) and pancreas cancer line cell (HPC3) was investigated by RT-PCR. cDNAs were generated by total RNAs derived from cell lines. *GAPDH* was used as an internal positive control. **C. SMCP expression profiles in human lung cancer tissues.** *SMCP* mRNA expression in human lung cancer tissues was investigated by RT-PCR. #1 and #2 are adenocarcinoma cases. #3 and #4 are squamous cell carcinoma cases. #5 is a large cell carcinoma case. T indicates lung cancer tissue. N indicates adjacent lung normal tissue. *GAPDH* was used as an internal positive control. **D. Schema of novel variant form of SMCP.** SMCP wild type (variant 1) is composed of exon 1 and exon 2. The novel isoform of SMCP (variant 2) has only one exon with a 5′ terminal additional extension. **E. Expression profiles of SMCP variant 1 and variant 2 in the testis and cancer cells.** SMCP mRNA expression was investigated by RT-PCR using an F1 and F2 mixture primer as a forward primer and R1 primer as a reverse primer in testis and cancer cells. Variant 1 was a 152-bp PCR product, and variant 2 was a 624-bp PCR product. **F. Expression of SMCP protein.** Detection of SMCP protein in HEK293T cells transfected with expression vectors of variant1 CDS and variant2 as assessed by Western blot analysis with polyclonal SMCP antibody. Beta-actin was used as a protein loading control.

RT-PCR analysis was performed to determine the expression of *SMCP* in human fetal and adult normal tissues and in cancer cells. *SMCP* mRNA was expressed only in the testis at a high level among human adult tissues (Figure 2A). *SMCP* mRNA was detected in all cancer cells tested in this study, including renal cell carcinoma cells (Caki-1, ACHN, SMKT-R1, SMKTR-2, SMKTR-3 and SMKTR-4), lung squamous cell carcinoma cells (Sq-1), lung small cell carcinoma cells (Lc817), lung large cell carcinoma cells (86-2 and Lu99), lung adenocarcinoma cells (1-87 and A549) and pancreas carcinoma cells (HPC3) (Figure 2B). *SMCP* mRNA was preferentially expressed in primary lung cancerous tissues in 5 cases (2 adenocarcinomas, 2 squamous cell carcinomas and 1 large cell carcinoma) than those in adjacent normal counterpart lung tissues (Figure 2C).

### Novel SMCP splicing variant2 is the dominant form in cancer cells

The SMCP transcript is composed of two exons (Variant 1, Figure 2D upper panel), and we tried to detect the SMCP transcript by quantitative PCR (qPCR) using a probe designed at the junction of exon 1 and exon 2. We detected *SMCP* mRNA in the testis tissue, but we did not detect *SMCP* mRNA in any of the cancer cells by qPCR. We therefore hypothesized that the transcript of SMCP in cancer cells was different from that in the testis. To address the structure of *SMCP* mRNA, we performed 5′ RACE and 3′ RACE. The cDNA template used was prepared from the Lc817 cell line, which showed a high level of *SMCP* transcript (Figure 2B). The 5′ RACE PCR amplification revealed that the *SMCP* mRNA from Lc817 is composed of only one exon that is overlapped with exon 2 with 5′ extension (termed SMCP variant 2) and shares coding sequence (CDS) (Figure 2D). We therefore analyzed the SMCP mRNAs in testis and cancer cells using a mixture of SMCP variant1-specific primer (F1) and SMCP variant2-specific primer (F2), and we found that SMCP vt1 is expressed in the testis and that SMCP vt2 is expressed in cancer cells (Figure 2E). We could detect a specific band with anti-SMCP polyclonal antibody in the HEK293T cell line transfected with expression vectors of both variant2 and variant1 CDS (Fig. 2F), suggesting that both variant1 and variant2 have the same protein products. We therefore used CDS expression constract for further analyze of SMCP functions.

### SMCP has a role in tumor-initiating ability

**Figure 3:**
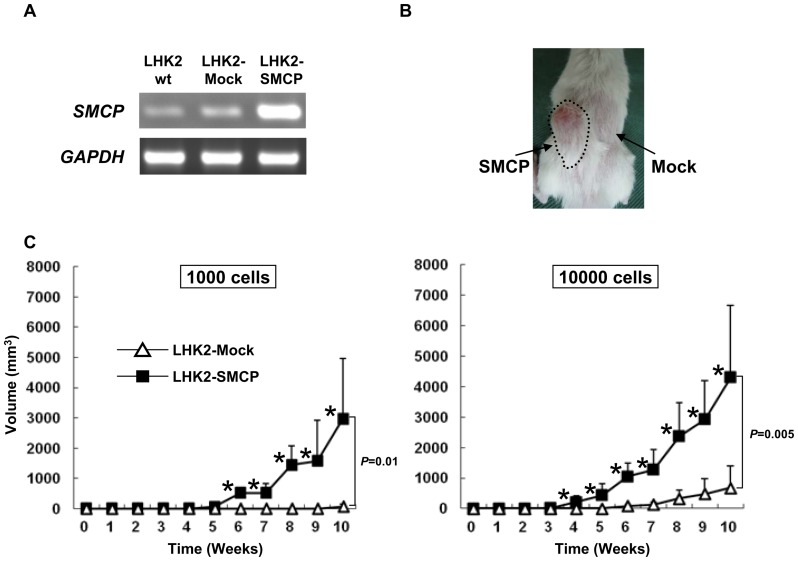
SMCP has a role in the tumor-initiating ability in LHK2 lung adenocarcinoma cells. **A. Expression of SMCP in SMCP-overexpressed LHK2 cells.** Expression of SMCP in LHK2-SMCP cells was confirmed by RT-PCR. **B. Representative picture of tumors injected with SMCP-overexpressed LHK2 cells. C. Tumor growth of LHK2-SMCP cells and LHK2-Mock cells.** One×10^3^ and 1×10^4^ of LHK2-SMCP cells and LHK2-Mock cells were injected into NOD/SCID mice subcutaneously. Data are means + SD. Asterisks represents statistical significant difference. Student's t-test. *P*<0.05.

Since SMCP is preferentially expressed in SP cells, we further analyzed the function of SMCP using SMCP-overexpressing cells. SMCP cDNA was stably transduced into LHK2 cells, and its expression was confirmed by RT-PCR (Figure 3A). A tumor was initiated by 1×10^2^ LHK2-SMCP cells in 1 of 5 mice, and tumors were initiated by 1×10^3^ and 1×10^4^ LHK2-SMCP cells in 3 of 5 mice and 5 of 5 mice, respectively. On the other hand, a tumor was initiated by 1×10^4^ LHK2-Mock cells in 3 of 5 mice, and no tumor initiation was observed with 1×10^2^ or 1×10^3^ LHK2-Mock injection (Table 2). Furthermore, the growth of tumors derived from LHK2-SMCP cells was significantly faster than that of tumors derived from LHK2-Mock cells (Figure 3B and 3C).

**Table 2:** Tumor-initiating ability of LHK2-SMCP and LHK2-mock.

		Cell numbers of injection into NOD/SCID mice			
Day 35 post injection		1×10^1^	1×10^2^	1×10^3^	1×10^4^
LHK2-SMCP	Incidence	0/5	1/5	3/5	5/5
	Volumes (mm^3^)	-	4.0	57.3±43.9	436.3±386.2*
LHK2-mock	Incidence	0/5	0/5	0/5	3/5
	Volumes (mm^3^)	-	-	-	4.83±7.5

Incidence indicates the number of tumor formation/number of injections.

Tumor volumes are mean±S.D.

* P<0.01, compared with tumor volumes of 1×10^4^ Mock cells.

LHK2-SMCP, SMCP-overexpressing LHK2 cells; LHK2-mock, mock-transfected cells.

### Downregulation of SMCP by siRNA abrogates tumor-initiating ability of wild-type and SP cells of lung cancer cells

**Figure 4:**
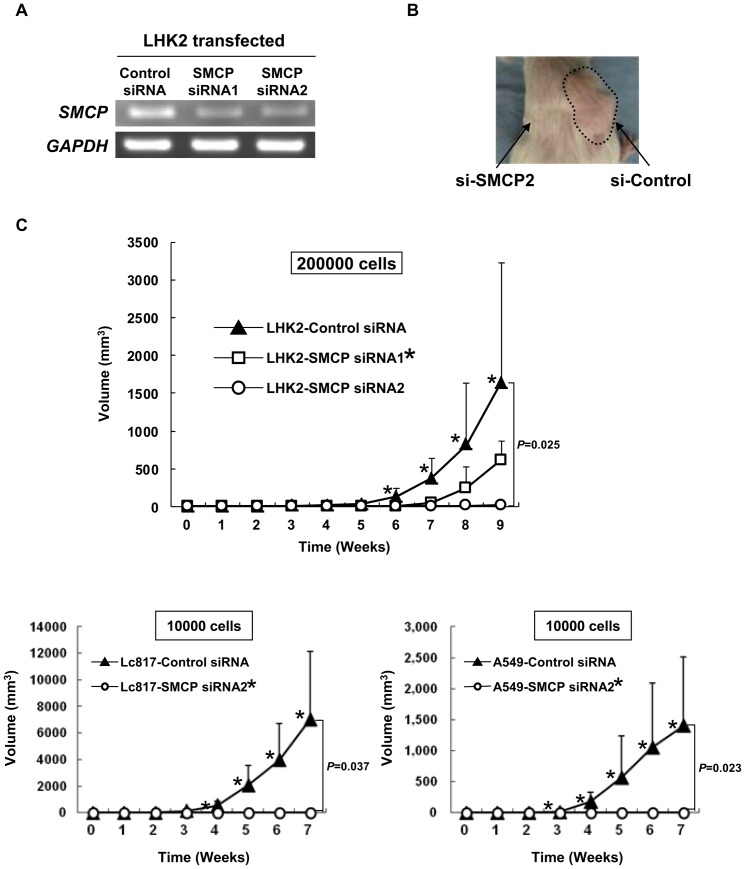
SMCP knockdown abrogates tumor initiation of LHK2 cells. A. Downregulation of SMCP expression in LHK2 cells transfected with siRNA. B. Representative picture of tumors injected with SMCP knockdown LHK2 cells. C. Tumor growth of LHK2, A549 and Lc817 cells transfected with SMCP siRNAs. Two×10^5^ LHK2 cells transfected with control siRNA or SMCP siRNA 1 and 2 were injected into NOD/SCID mice subcutaneously. One×10^4^ A549 cells transfected with control siRNA or SMCP siRNA2 were injected into NOD/SCID mice subcutaneously. One×10^4^ Lc817 cells transfected with control siRNA or SMCP siRNA2 were injected into NOD/SCID mice subcutaneously. Data are means + SD. Asterisks represents statistical significant difference. Student's t-test. *P*<0.05.

The results obtained by using SMCP-overexpressing cells suggested that SMCP has a role in tumor initiation, and we therefore performed a gene knockdown study using siRNAs. We designed two different siRNAs and confirmed gene knockdown by RT-PCR using SMCP siRNA-transfected LHK2 cells (Figure 4A). To investigate the tumor-initiating ability of SMCP knockdown cells, we injected SMCP siRNA-transfected LHK2, Lc817 and A549 cells into NOD/SCID mice. Tumor growth was significantly suppressed in mice injected with SMCP siRNA1 and siRNA2-transfected LHK2, Lc817 and A549cells compared to that in mice injected with control siRNA-transfected LHK2 cells (Figure 4B and 4C and table 3).

**Table 3:** Tumor-initiating ability of SMCP siRNA and Control siRNA.

LHK2		Cell numbers of injection into NOD/SCID mice		
Day 35 post injection		Contorl siRNA	SMCP siRNA1	SMCP siRNA2
2×10^5^	Incidence	6/6	2/5	0/5
	Volumes (mm^3^)	27.8±15.7	0.9±1.7*	-

Lc817		Cell numbers of injection into NOD/SCID mice		
Day 28 post injection		Contorl siRNA	SMCP siRNA1	SMCP siRNA2
1×10^4^	Incidence	3/4	-	0/4
	Volumes (mm^3^)	541.1±299.2	-	0.0±0.0†

A549		Cell numbers of injection into NOD/SCID mice		
Day 28 post injection		Contorl siRNA	SMCP siRNA1	SMCP siRNA2
1×10^4^	Incidence	4/4	-	0/4
	Volumes (mm^3^)	176.6±147.4	-	0.0±0.0†

Incidence indicates the number of tumor formation/number of injections.

Tumor volumes are mean±S.D.

* P<0.01, compared with tumor volumes of 2×10^5^ Control siRNA cells.

† P<0.05, compared with tumor volumes of 1×10^4^ Control siRNA cells.

**Figure 5:**
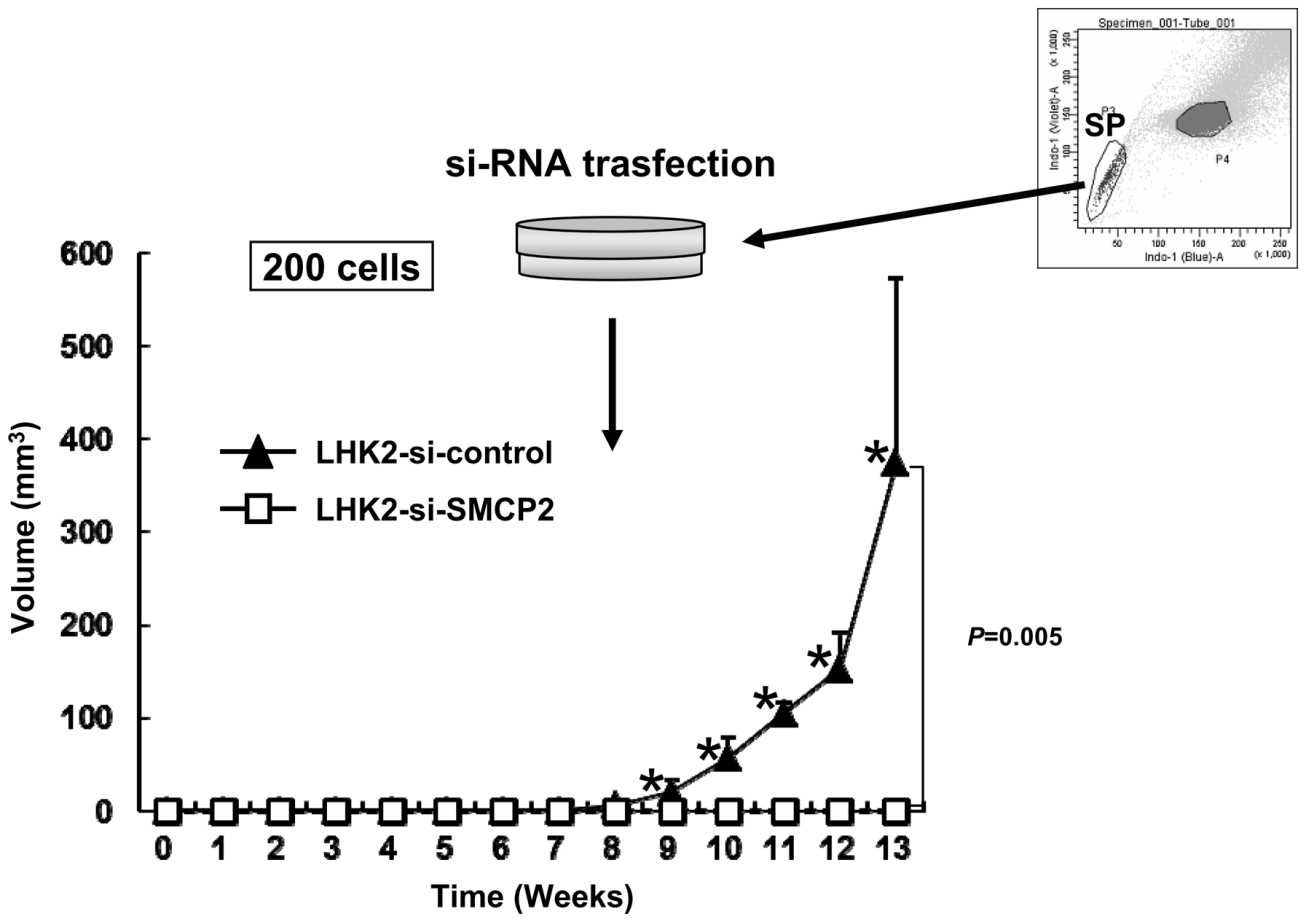
SMCP knockdown abrogates tumor initiation of LHK2 SP cells. LHK2 SP cells were isolated and seeded onto a 24-well plate. SMCP siRNA was transfected into LHK2 SP cells. Two days after transfection, LHK2 SP cells were injected into NOD/SCID mice. Data are means + SD. Asterisks represents statistical significant difference. Student's t-test. *P*<0.05.

Since *SMCP* was preferentially expressed in SP cells, we therefore performed gene knockdown using SP cells derived from wild type LHK2 cells. Two hundred siRNA-transfected LHK2 SP cells were injected into the backs of NOD/SCID mice. Control siRNA-transfected LHK2 SP cells initiated tumors, whereas SMCP siRNA-transfected LHK2 SP cells did not initiate tumors (Figure 5). Taken together, these results indicate that SMCP has a role in the tumor-initiating ability of CSCs/CICs.

## Discussion

In this study, we investigated the gene expression profile of SP cells derived from LHK2 lung adenocarcinoma cells. The existence of CSCs/CICs has been discussed widely over the past decade [40], [41] [42]. The concept of stem cell-like tumor cells is intriguing; however, identification and isolation of such cells is difficult. Stem cells are identified by expression of surface markers or functional markers such as ABC transporters or ALDH, and clonogenic potential and capacity to regenerate tumors of the identified subset of tumor cells are subsequently demonstrated [42]. Although the field of CSC/CIC biology is a relatively new field, continued elucidation of the features of these cells holds promise for the development of novel patient therapies [42] [43]. Moreover, evidence supporting the existence of CSCs/CICs in various solid tumors has been obtained; however, a definite CSC/CIC marker has not yet been established. Attempts to enrich CSCs/CICs by using SP analysis have been made in several studies [10], [35], [38], [44]–[47]. In this study, we succeeded in isolating SP cells from LHK2 lung carcinoma cells, SW480 colon carcinoma cells and MCF7 breast carcinoma cells. SP cells exhibited greater tumor-initiating ability and higher expression levels of iPS cell-related genes (SOX2, POU5F1 and NANOG), indicating that SP cells derived from LHK2, SW480 and MCF7 cells are enriched with CSCs/CICs and are a reasonable source for further research.

Gene expression profiling using cDNA microarrays and RT-PCR analysis revealed that the sperm-related gene *SMCP* is ectopically expressed in SP cells derived from LHK2, SW480 and MCF7 cells. Several molecular mechanisms of cancer stem cells have been described. Weinberg's group described that breast cancer stem cells also have epithelial-mesenchimal transition (EMT) phenotypes [48]. However, we did not detect any EMT related genes by cDNA microarray. SOX2 have been showed to be related to lung carcinogenesis, and we also found that SOX2 was overexpressed in LHK2 SP cells [49] [50] [38]. Other report showed that cytosolic glycine decarboxylase was related to the tumor-initiating ability of lung cancers [9]. However, this is the first report that mitochondria-related gene has tumorigenic potential.

The product of the *SMCP* gene localizes to the capsule associated with the mitochondrial outer membranes and is thought to function in the organization and stabilization of the crescent structure of the sperm's mitochondrial sheath [41]. Sperm mitochondria differ in morphology and subcellular localization from those of somatic cells. They are elongated, flattened, and arranged circumferentially to form a helical coiled sheath in the midpiece of the sperm flagellum. In this study, we investigated the distributions of *SMCP* protein, and we found that GFP-fused SMCP protein is expressed in the mitochondria of lung carcinoma cells. Since SMCP is expressed in the testis and cancer cells, SMCP is a novel cancer-testis (CT) gene [51]. The testis is also an immune-privileged site [52]. Therefore, CT antigens are highly immunogenic and are promising targets for cancer immunotherapy [53]–[55]. Some cancer–testis antigens have been isolated by analyzing a testis cDNA expression library with cancer patients' sera [54]. Although *SMCP* has not been reported by screening using cancer patients' sera, SMCP was reported to be recognized by sera from rats immunized by sperm [56]. Thus, SMCP might be immunogenic to the humoral immune system. Indeed, we could detect anti-SMCP antibody in cancer patients' sera by an ELISA assay using SMCP recombinant protein (unpublished data). Anti-SMCP antibody might therefore be a new useful biomarker for detection of CSCs/CICs that is related to prognosis of cancer patients.

RACE and RT-PCR analysis revealed that the transcript of SMCP in CSCs/CICs was a variant form (SMCP vt2). SMCP vt2 lacks exon 1 and has only one exon 2 which has 889 base pair extension to the 5′-end. Since wild-type SMCP (SMCP vt1) and SMCP vt2 share the same coding sequence, the SMCP protein structures in CSCs/CICs and the testis should be same. However, the transcriptional start point of SMCP vt2 is approximately 6000 base pairs downstream of the transcriptional start point of SMCP vt1. Thus, the transcription factor and promoter that are responsible for the transcription of SMCP vt 2 should be different from those of SMCP vt 1. Further molecular analysis is needed.

In this study, overexpression of SMCP enhanced tumorigenicity *in vivo*. Moreover, transient knockdown of *SMCP* mRNA by SMCP-specific siRNA completely abrogated the tumorigenicity of LHK2 bulk cells and even SP cells. These observations indicate that SMCP has a pivotal role in tumorigenicity of lung cancer; however, its exact molecular mechanisms are still elusive.

In summary, we identified a novel variant form of the sperm-specific antigen *SMCP* that is expressed in CSCs/CICs and showed that *SMCP* plays a role in lung CSC/CIC tumorigenicity. Since *SMCP* is expressed in cancer tissues but not in normal tissues except for the testis, *SMCP* might be a novel CSC/CIC marker and a promising and potential target of CSC/CIC-targeting therapy.

## Supporting Information

Table S1 **Summary of upregulated genes in LHK2 SP cells.** The summary of upregulated genes in SP cells derived from LHK2 cells. Cy5/Cy3: SP cells were labeled with Cy5 and MP cells were labeled with Cy3. Cy3/Cy5: SP cells were labeled with Cy3 and MP cells were labeled with Cy5.
(XLS)
